# Spleen Contraction During Sudden Eupneic Hypoxia Elevates Hemoglobin Concentration

**DOI:** 10.3389/fphys.2021.729123

**Published:** 2021-09-21

**Authors:** Frank Pernett, Felix Schagatay, Caroline Vildevi, Erika Schagatay

**Affiliations:** ^1^Department of Health Sciences, Mid Sweden University, Östersund, Sweden; ^2^Scandinavian Aviation Academy, Västerås, Sweden; ^3^Swedish Winter Sports Research Centre, Östersund, Sweden

**Keywords:** extreme environment, ultrasound, acute survival, high altitude, arterial oxygen saturation

## Abstract

The spleen contracts progressively during moderate normobaric hypoxia exposure of 20 min, which elevates hemoglobin concentration (Hb). However, acute hypoxia exposure could be shorter and more severe when oxygen systems fail during, e.g., high-altitude sky diving, aircraft cabin pressure drop, balloon flights, extreme altitude climbing, and in some maladies. We aimed to evaluate the speed and magnitude of spleen contraction during short exposure to extreme eupneic hypoxia and its subsequent recovery on oxygen. Eight female and seven male volunteers were exposed to normobaric hypoxia (10% oxygen) for 10 min during sitting rest, followed by 10 min on 100% oxygen. Heart rate (HR), arterial oxygen saturation (SpO_2_), and mean arterial blood pressure (MAP) were measured continuously. The spleen was measured via ultrasonic imaging every minute for volume calculations, and venous blood samples were drawn before and after exposure for hemoglobin concentration (Hb). Mean (SD) spleen volume was 279 (115) mL before exposure, 219 (75) mL (21% reduction; *P* = 0.005) at 3 min of exposure, and 201 (93) mL after 10 min exposure to hypoxia (28% reduction; *P* < 0.001). Hb was 138.8 (7.6) g·L^−1^ before and 142.9 (8.1) g·L^−1^ after 10 min of exposure (2.9% increase; *P* < 0.001). SpO_2_ was 96.4 (1.7)% before exposure and 74.7 (8.4)% during the last minute of exposure (22.5% reduction; *P* < 0.001). HR increased from 80 (14) to 90 (17) bpm during exposure (12% increase, *P* < 0.05). MAP remained unchanged. After 10 min recovery on oxygen, values had been restored for spleen volume and Hb, while SpO_2_ was higher and HR lower compared with before hypoxia exposure. We concluded that acute normobaric hypoxia of only 10 min caused significant spleen volume contraction with Hb increase. This rapid spleen response, evident already after 3 min of exposure, could have a protective effect during sudden exposure to severe hypoxia.

## Introduction

Sudden decreases in oxygen partial pressure can occur in an abrupt loss of cabin pressure in an aircraft (Johnston, 2008; Muehlemann et al., 2013), failure of the oxygen delivery system in high-altitude parachuting (Clemente-Suárez et al., 2017; Ottestad et al., 2017), high-altitude ballooning (Pilmanis and Sears, 2003), or climbing (West, 2003). This is quite different from chronic exposure where acclimatization plays an important role and also from gradually occurring mild hypoxia. Sudden hypoxemia can also occur in illnesses as acute pulmonary embolism (Fernandes et al., 2019). Another situation is apneic hypoxia, which is associated with several specific responses (Schagatay, 2009) but differs from eupneic hypoxia as it implies the cessation of normal respiration.

The physiological response to acute eupneic hypoxia includes increases in rate and depth of breathing (Cummins et al., 2020), increased heart rate, higher pulmonary vascular resistance, and a reduction in plasma volume (Luks et al., 2021). However, the acute responses to hypoxia are far from fully explained, and several mechanisms contribute to the outcome on the cellular level. The spleen is known for its immunological properties, but it has a variety of functions and can acutely reduce its volume, eliciting increases in hemoglobin concentration (Hb), in response to hypoxia and various stressful stimuli (Stewart and McKenzie, 2002). Hypoxia induced by apnea is associated with elevated Hb, a response not found in splenectomized subjects (Schagatay et al., 2001; Baković et al., 2003). This is an active response (Baković et al., 2003) that has also been observed during high altitude exposure, where the spleen reduces its volume even more with exercise (Sonmez et al., 2007; Engan et al., 2014; Schagatay et al., 2020b). Functional importance is indicated by the observation that spleen volume was negatively associated with symptoms of acute mountain sickness on the ascent in lowlanders (Holmström et al., 2019) and that spleen volume was larger in Sherpa highlanders, especially in those still living on high altitude, compared with lowlanders (Holmström et al., 2020).

There is also evidence of spleen contraction in other conditions associated with cellular hypoxia, like abdominal blunt trauma (Cruz-Romero et al., 2016), severe acute respiratory syndrome (Ding et al., 2003), and drowning (Haffner et al., 1994). Even when there is relative cellular hypoxia due to increased oxygen consumption, the spleen reduces its volume during heavy exercise (Laub et al., 1993; Shephard, 2016; Schagatay et al., 2020b) and during mild exercise like walking in individuals with pulmonary disease (Schagatay et al., 2015). Thus, the spleen seems to have a general role as a potentially protective mechanism against hypoxia, but it has never been studied if and how soon the spleen response occurs during sudden severe eupneic hypoxia.

When the spleen contracts, it releases erythrocytes into the systemic circulation increasing the Hb and oxygen content in the blood (Espersen et al., 2002). The spleen contraction is also evident during moderate normobaric hypoxia as short as 20 min (Richardson et al., 2008; Lodin-Sundström and Schagatay, 2010) where exposure to 12.8% and 14.2% of oxygen evoked spleen contractions of 18% and 16%, respectively. Hypoxia thus seems to be the main trigger of spleen contraction (Richardson et al., 2008) but it was found that eupneic hypoxia for 20 min did not evoke the same magnitude of spleen contraction as did apnea-induced hypoxic hypoxia of the same severity (Lodin-Sundström and Schagatay, 2010), leading to the suggestion that either the associated hypercapnia, the rate of desaturation, or the apnea input itself was involved. It was later shown that the spleen also responded to hypercapnia (Richardson et al., 2012), while the two other inputs have not been further studied.

In activities done at extreme altitudes over 5.500 m, failure of oxygen supply can suddenly expose the body to severe hypoxemia, with a rapid drop in SpO_2_. This hypoxia exposure during high-altitude sky diving, aircraft cabin pressure drop, and balloon flights resembles that seen in some maladies and can be shorter and more severe than those studied previously. It is unclear if the spleen response is occurring rapidly enough to make a difference in these situations, and if it evokes an elevation in Hb.

We, therefore, aimed to evaluate the speed and magnitude of spleen contraction and its possible effects on Hb during short sudden exposure to extreme hypoxia and its subsequent recovery on oxygen. We hypothesized that the spleen will contract and elevate Hb under acute severe hypoxia as short as 10 min. We further hypothesized that the response will develop within minutes and resolve within a few minutes on oxygen.

## Materials and Methods

### Participants

Volunteers were 15 adults, eight females and seven males, with a mean (SD) age of 25 (3) years, height 177 cm (males 185, females 168 cm), and weight 76 kg (males 87, females 66 kg). The participants were all healthy non-smokers involved in physical training 6.6 (3.5) h per week. None of the participants had been at an altitude above 2500 m in the past 6 months. The participants received detailed information about the test protocol and signed an informed consent form. The protocol had been approved by the Regional Human Ethics Board of Umeå University, Sweden, and complied with the Helsinki declaration.

### Protocol

The participants arrived at the lab after a period of 12 h without any alcohol consumption and 2 h without eating or drinking caffeine-containing beverages before the testing started. Height and weight were measured, and vital capacity was measured in the standing subject in triplicate with the largest volume used (Spirolite 201 spirometer, Vise Medical Co. Ltd, Chiba, Japan). The participant was seated and had a venous catheter (BD Venflon™ Pro, Becton Dickinson Infusion Therapy AB, Helsingborg, Sweden) inserted in an antecubital vein of the left arm after which all other measurement equipment was attached. The protocol, which started after at least 20 min of sitting rest, consisted of three phases: (1) 5 min of normobaric normoxic respiration (PRE), (2) 10 min of normobaric hypoxic respiration of gas containing 10.0% oxygen in nitrogen (Hypoxico, Hypoxico Inc., New York, NY, USA) equivalent to 5,793 m altitude (EXP), and (3) 10 min of normobaric hyperoxic respiration (POST) at 100% oxygen, mimicking rescue oxygen breathing. The different gas mixtures were administered via face mask.

### Measurements

Minute-to-minute measurements of spleen size were obtained via ultrasonic imaging (Mindray DP-6600, Shenzhen Mindray Bio-Medical Electronics CO., Ltd., Shenzhen, China) where three-axial spleen diameters were measured. Blood samples (3 mL) were drawn during the last minute of each phase and analyzed directly for Hb in triplicate (ABx Diagnostics Micros 60 CT, Montpellier, France). In two participants, capillary samples were taken in triplicate, as catheter insertion was not possible, and Hb was analyzed with HemoCue (HemoCue [Hb] 201+, HemoCue AB, Ängelholm, Sweden).

Heart rate (HR) and peripheral oxygen saturation (SpO_2_) were measured continuously across all phases with a pulse oximeter with a finger probe (Biox 3700e, Ohmeda, Madison, USA), and mean arterial blood pressure (MAP) was measured with a photoplethysmometer via finger cuff placed on the right hand (Finapress 2300, Ohmeda, Madison, USA). The end-tidal oxygen concentration (EtO_2_) and the end-tidal carbon dioxide concentration (EtCO_2_) in the face mask were measured via a gas analyzer (NormocapOxy, Datex Ohmeda, Helsinki, Finland). To avoid hypoxic syncope in the participants, the minimum SpO_2_ accepted was set at 65%; if the value was reached, phase 2 was terminated, and the participant was moved to phase 3. Continuous data were stored in a computer, together with an event-marker, via a BioPac MH100A CE multichannel data acquisition system (Biopac Systems Inc., Goleta, CA, USA) and were analyzed further using AcqKnowledge Software (Biopac Systems Inc, USA). Breath-by-breath data were analyzed for respiratory frequency and inhaled and exhaled oxygen fraction.

### Analysis

Spleen volume was calculated with the Pilström equation: $L\times \pi \left(\frac{\left(W\times T-T^{2}\right)}{3}\right)$ using the spleen maximal length (L), width (W), and thickness (T) obtained from the ultrasound measurements (Schagatay et al., 2005). This formula describes the differences between two ellipsoids divided by two, based on a generalized spleen shape. The spleen volume of the individual in the resting normobaric state before exposure was calculated based on the averaged measures during the last 3 min before the start of hypoxia and used as baseline spleen volume. End of exposure spleen volume was the average of the last 3 min of EXP. Recovery spleen volume was the average of the last 3 min of POST.

### Statistical Analysis

Participants served as their own controls. The normal distribution of all data was tested using the Shapiro-Wilk test. Paired Student's *t*-test was used to compare PRE and EXP gas values. Assessments of interaction effects between the independent variables (treatment [PRE, EXP; POST]) and sex on the dependent variables (spleen volume, Hb, SpO_2_, and HR) were conducted by a two-way mixed ANOVA with Bonferroni corrections for repeated measures. Assessments of associations between dependent variables were conducted with bivariate correlation tests using Pearson product-moment correlation coefficient (*r*). Meaningfulness of effects was estimated by the standardized mean difference (Cohen's *d*, effect size [ES]) computed as the mean difference divided by the pooled SD. ES was presented along with 95% confidence intervals [CI]. Statistically significant difference was accepted at *P* < 0.05. Interpretation of ES was based on three categories: 0.0–0.5 was considered a small effect, 0.6–1.1 was considered a medium effect, and ≥1.2 was considered a large effect.

## Results

All participants completed the study protocol. In one participant, due to an unusual shape of the spleen, measurements were not possible, and in another participant, Hb samples could not be obtained. These two participants were withdrawn from the respective analysis and entirely from the correlation analysis. Results are presented as mean ± standard deviation (SD) with *n* indicated for the respective variable.

Males had a larger vital capacity and a larger pre-exposure spleen volume than females but Hb baseline levels were similar between sexes (Table 1). The two-way mixed ANOVA revealed a significant treatment effect on all dependent variables (spleen volume, Hb, SpO_2_, and HR). There was no two-way interaction effect between males and females on the dependent variables during the experimental intervention, showing that these variables change equally between sexes in response to the hypoxia. Results are therefore presented as mean (SD) for the whole group.

**Table 1:** Baseline characteristics divided by sex.

	**Male**	**Female**	***P***
**Characteristics**			
Vital capacity (L)	6.1 (0.5)	4.1 (0.6)	<0.001
Hb (gr.L^−1^)^*^	142.8 (8.7)	137.0 (6.7)	0.394
Spleen volume (mL)^*^	353 (104)	204 (71)	0.018

*Units: gr.L^−1^ (grams per liter), mL (mililiters)*.

* *n = 14*.

### End-Tidal Gas values

The EtO_2_ was 98.6 (17.2) mmHg at PRE, and it decreased to 46.5 (6.3) mmHg during EXP (*P* < 0.001; ES = 4.00 [3.26–4.75]). The EtCO_2_ was 32.7 (4.3) mmHg at PRE, and it decreased to 28.3 (4.7) mmHg during EXP (*P* < 0.001; ES = 0.98 [0.23–1.72]).

### Oxygen Saturation

**Figure 1:**
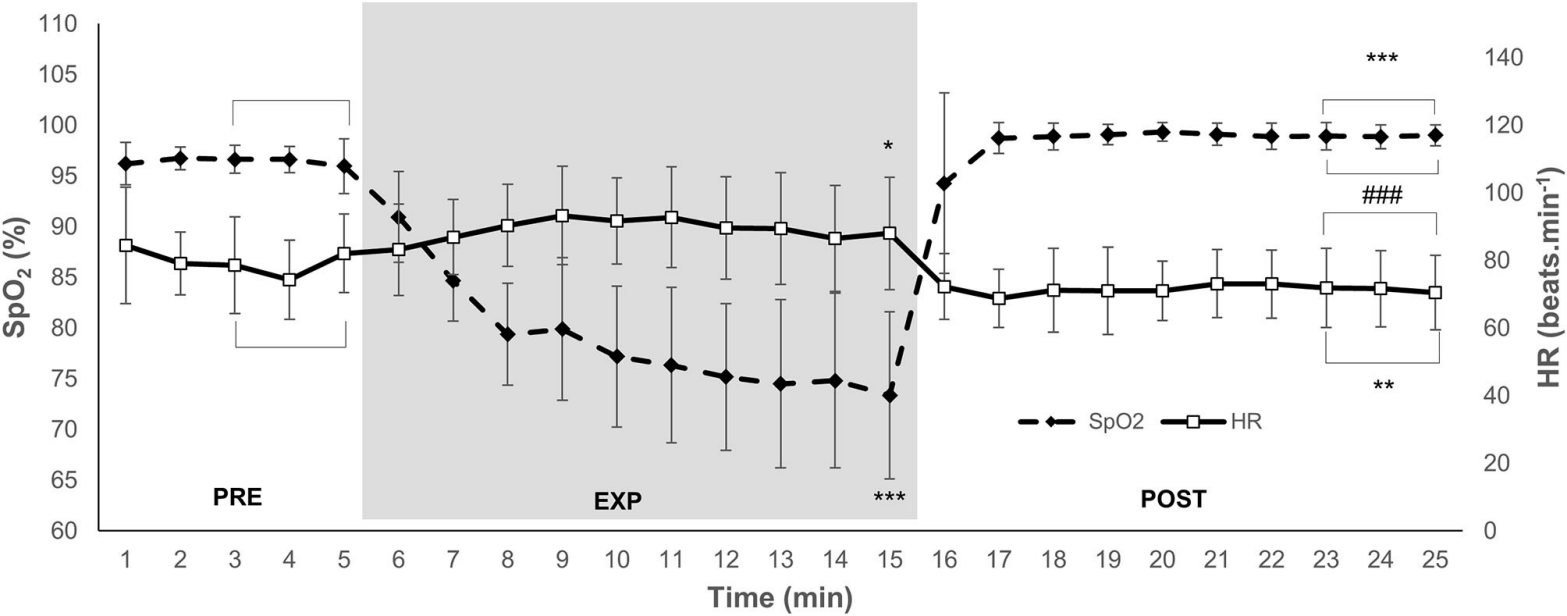
Mean and SD arterial oxygen saturation (SpO_2_) and heart rate (HR) during 5 min normoxic breathing (PRE), 10 min hypoxic breathing of 10% O_2_ (EXP), and 10 min hyperoxic breathing of 100% O_2_ (POST) for *n* = 15. Gray zone indicates EXP. Horizontal brackets indicate an averaged value for that period. Significant difference from (PRE) is indicated by * for *P* < 0.05, ** for *P* < 0.01, and *** for *P* < 0.001. Significant difference from EXP is indicated by ### for *P* < 0.001.

The SpO_2_ was 96.4 (1.7)% during PRE, and it decreased to 74.7 (8.4)% by the end of EXP (*P* < 0.001; ES = 3.94 [3.20–4.69]; Figure 1). During the last 3 min of POST, SpO_2_ increased to 98.8 (1.1)% (*P* < 0.001; ES = 4.56 [3.81–5.31]), which was also higher compared with the PRE value (*P* < 0.001; ES = 1.90 [1.15–2.65]; Figure 1).

### Cardiovascular Parameters

The HR was 80 (14) beats·min^−1^ during PRE, and it increased to 90 (17) beats·min^−1^ in EXP (*P* = 0.046; ES = 0.64 [−0.11–1.39]; Figure 1). The HR decreased to 72 (9) beats·min^−1^ during POST (*P* < 0.001; ES = 1.32 [0.58–2.07]), which was also lower compared with PRE (*P* = 0.002; ES = 0.68 [−0.07–1.43]; Figure 1). The MAP was 111 (21) mmHg during PRE. There was no change in MAP during EXP at 113 (33) or POST at 111 (19) mmHg (NS).

### Spleen Volume

**Figure 2:**
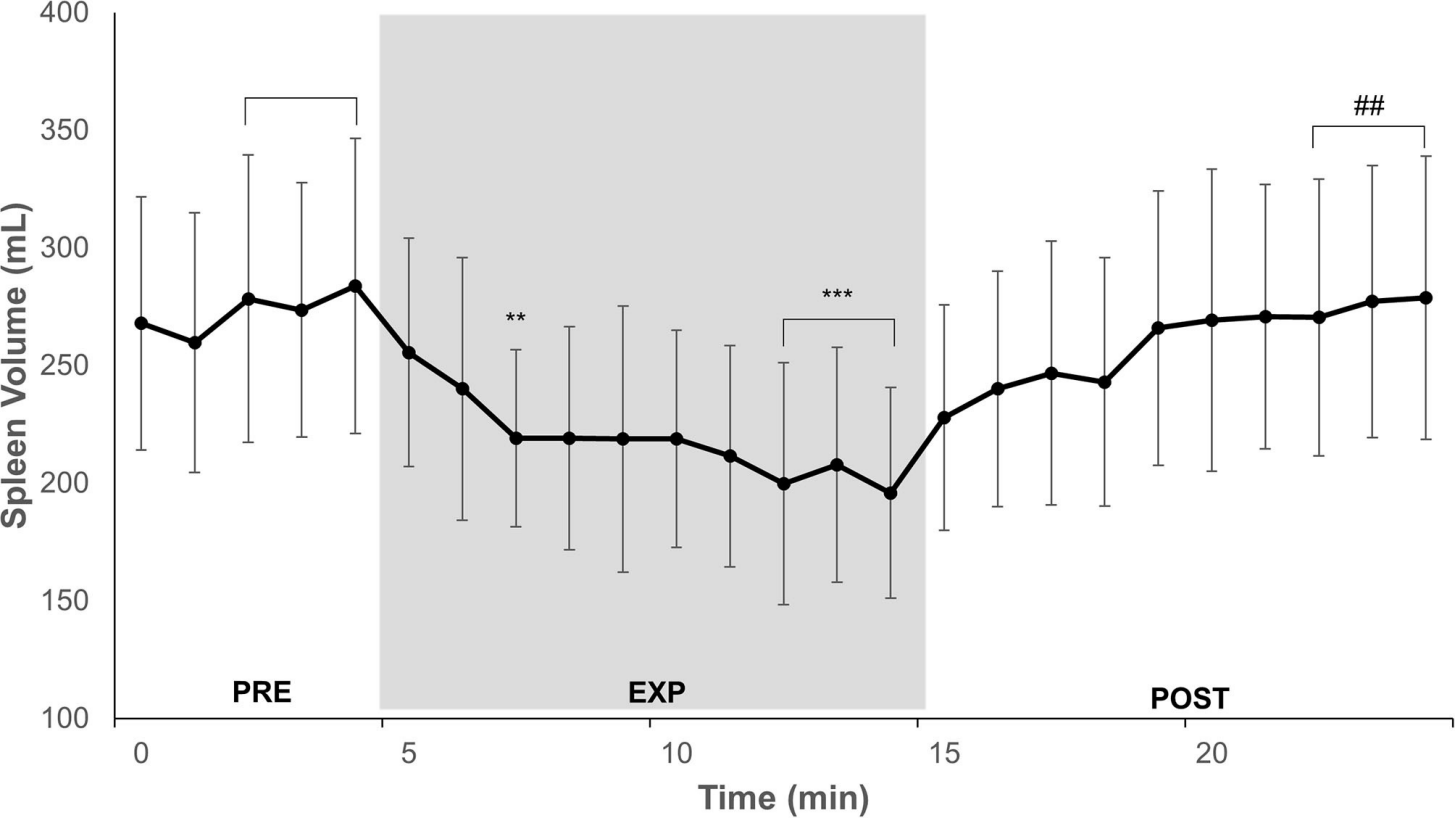
Minute-to-minute mean and SD of spleen volume during 5 min normoxic breathing (PRE), 10 min hypoxic breathing of 10% O_2_ (EXP), and 10 min hyperoxic breathing of 100% O_2_ (POST) for *n* = 14. Gray zone indicates EXP. Horizontal brackets indicate an averaged value for that period. Significant difference from PRE is indicated by ** for *P* < 0.01 and *** for *P* < 0.001. Significant difference from EXP is indicated by ## for *P* < 0.01.

Spleen volume for 14 participants during PRE was 279 (115) mL. After 3 min of EXP, the spleen contracted to 219 (75) mL (by 21%; *P* = 0.005; ES = 0.62 [−0.16–1.39]), and during the last 3 min of EXP, the volume decreased to 201 (93) mL (by 28%; *P* < 0.001; ES = 0.75 [−0.03–1.52]; Figure 2). At the end of POST, the spleen volume increased from EXP to 279 (120) mL (with 37%; *P* = 0.002; ES = 0.73 [−0.05–1.50]), which was the same as the PRE value (*P* = 1.000; ES = 0.00 [−0.78–0.78]; Figure 2). The individual spleen volume reduction from PRE to EXP ranged between 0 and 134 mL.

### Hemoglobin Concentration

**Figure 3:**
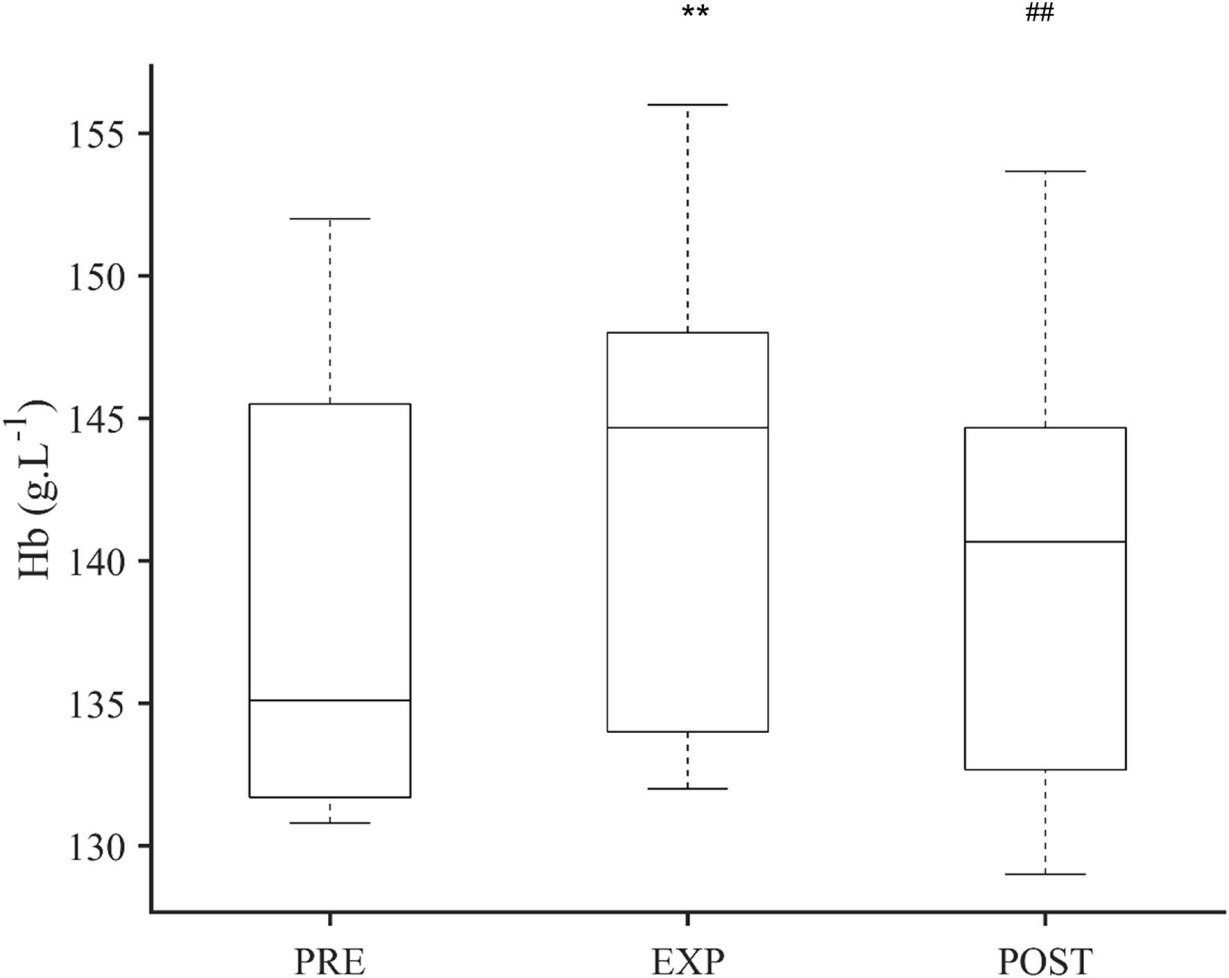
Boxplot of hemoglobin concentration (Hb) after 5 min normoxic breathing (PRE), 10 min hypoxic breathing of 10.0% O_2_ (EXP), and 10 min hyperoxic breathing of 100% O_2_ (POST) for *n* = 14. Significant difference from PRE is indicated by ** for *P* < 0.01 and from EXP is indicated by ## for *P* < 0.01. There was no difference between PRE and POST.

The Hb for 14 participants at PRE was 138.8 (7.6) g·L^−1^, and after EXP, it was 142.9 (8.1) g·L^−1^, which was 4.1 g·L^−1^ higher than PRE (*P* < 0.001; ES = 0.52 [−0.26–1.30]; Figure 3). Between EXP and 10 min POST, Hb had decreased with 2.7 g·L^−1^ to 140.2 (8.0) g·L^−1^ (*P* = 0.007; ES = 0.34 [−0.44–1.11]; Figure 3). The 10 min POST Hb value was similar to the pre-value (*P* = 0.734; ES = 0.18 [−0.60–0.96]; Figure 3). The individual Hb increase from PRE to EXP ranged between 0.2 g·L^−1^ and 12.3 g·L^−1^.

### Correlation Analysis

There was a positive correlation between baseline spleen volume and spleen volume contraction during EXP (*r* = 0.692, *P* = 0.006), but no correlation between initial spleen volume and relative volume change (Table 2). There was no correlation between SpO_2_ nadir and spleen volume contraction nor SpO_2_ nadir and relative change in spleen volume (Table 2).

**Table 2:** Correlation analysis.

**Variable 1**	**Variable 2**	***r***	***P***
HR peak	Hb Δ	0.762	0.002
Spleen	Spleen Δ	0.692	0.006
SpO_2_ Δ	Hb Δ	0.687	0.007
SpO_2_ nadir	Spleen %	0.445	0.111
Spleen	Spleen %	−0.262	0.366
SpO_2_ nadir	Spleen Δ	−0.204	0.483
Spleen Δ	Hb Δ	0.104	0.736

*Correlation analysis results for n = 13 participants. Initial spleen volume (Spleen). Spleen reduction from PRE to EXP (Spleen Δ). Spleen relative volume change expressed as a percentage from PRE to EXP (Spleen %). Hb increase from PRE to EXP (Hb Δ). SpO_2_ drop from PRE to EXP (SpO_2_ Δ). Pearson's correlation factor = r*.

We also found a positive correlation between maximal heart rate at EXP with Hb Δ (*r* = 0.762, *P* = 0.002) and also between SpO_2_ Δ and Hb Δ (*r* = 0.687, *P* = 0.007; Table 2). No correlation was found between spleen volume contraction and Hb increase (Table 2).

## Discussion

This study shows that during a short sudden exposure of 10 min to severe hypoxia, the spleen contracts and Hb increases, implying that even during short-term exposure the spleen plays a role in the physiological response to acute hypoxia. Indeed, just after 3 min of hypoxia, there was a spleen volume reduction of 21%, with a maximal response at 28% reached after 7 min, and the response stayed stable during the last 3 min of exposure. Thus, there was a biphasic spleen volume reduction with a faster reduction in the beginning followed by a more subtle reduction associated with the gradual reduction in SpO_2_. Such a biphasic spleen response has been reported previously in a study on long apneas (Lodin-Sundström et al., 2009), but it has never been observed previously after eupneic acute hypoxia, and shows that a situation of sudden exposure may induce an early stress-induced spleen contraction, followed by the development of a more powerful response when hypoxia develops. This is contrary to the interpretation done by Palada et al. (2007), which concluded that spleen contraction is induced during just the initial seconds of apnea but without the influence of hypoxia. We instead suggest that both the general sympathetic stimulus and hypoxia contribute to the response associated with hypoxic exposure, at least during eupneic exposure. As the subjects were sitting down and not performing any type of physical activity, hypoxia can be considered the most likely cause of these further adjustments. The spleen contraction had a close relation to the decrease in SpO_2_ and the increased heart rate. This suggests that the spleen contraction is an attempt to maintain oxygenation, via elevation of Hb, to sustain body functioning in conditions of sudden hypoxia. Hb elevation, albeit small in the whole group, was highly individual and varied between no effect to 9% increase, and in strong responders, it could be large enough to significantly facilitate oxygen delivery. Concerning the individual variation, it is important to notice that one participant did not have any change in spleen size or Hb.

The development of the spleen response was much faster in this situation with simulated 5,793 m exposure than with the exposure to 3,110 m as in the study by Lodin-Sundström and Schagatay (2010). The rapid response occurs within the “time of useful consciousness” (Yoneda et al., 2000) identified in pilots during rapid cabin pressure fall, or failing oxygen systems during flight, which suggests that the response could be useful to prolong conscious time and enable the victim to take action in various hypoxic situations.

The spleen contraction was reversed and Hb restored within 7 min of breathing 100% oxygen, showing that it is a transient response that is fully reversed within minutes when the hypoxic stimulus ceases. This rapid recovery is in line with previous studies of spleen recovery within 10 min after longer normobaric hypoxic exposure (Richardson et al., 2008; Lodin-Sundström and Schagatay, 2010) and within 8–9 min after serial apneas (Schagatay et al., 2005). The underlying mechanism behind the normalizing of Hb after a hypoxic challenge is not fully understood. We speculate that passive enlargement of the spleen with elongation of the contractile elements of the capsule occurs when the spleen red pulp is filled with blood and that a filtering effect leads to a higher Hb in the spleen than in the circulating blood.

The 28% spleen volume reduction in this study was greater in magnitude than the 16% (Lodin-Sundström and Schagatay, 2010) and 18% (Richardson et al., 2008) found in previous studies using milder eupneic hypoxia, suggesting that the severity of hypoxia during sudden exposure is important in determining the magnitude, and thus, it seems like the response is graded.

We used 10.0% oxygen in the inhaled gas, leading to 74.7% SpO_2_, which was lower than the 87.2% SpO_2_ seen at 14.2% oxygen (Lodin-Sundström and Schagatay, 2010), but higher than the 64.4% at 12.8% oxygen (Richardson et al., 2008), although the last study result can be explained by extreme desaturation in one participant. This might suggest that the splenic contraction is affected not only by the level and duration of exposure but also by the sudden drop in oxygen concentration, in line with the previous suggestion that the rate of desaturation during normobaric hypoxia can be a factor modifying spleen contraction (Lodin-Sundström and Schagatay, 2010). Stewart et al. (2003) reported that the splenic contraction was not affected by the duration of submaximal exercise but lacked information about SpO_2_ during the different durations of exercise. A SpO_2_ nadir that does not correlate with the decrease in spleen volume suggests that also other factors besides hypoxia may stimulate the contraction. Individual stress response and catecholamine levels play a role in spleen contraction (Stewart et al., 2003; Purdy et al., 2019), and according to the great HR variation during hypoxia in our participants, we can speculate that different individual levels of stress could have affected the spleen response. However, it is in line with previous studies that the spleen response magnitude on the same stimulus can be highly individual (Espersen et al., 2002; Prommer et al., 2007; Engan et al., 2014). The positive correlation that we found between peak HR and Hb increase is interesting, as sympathetic activation could be the common link.

Larger spleens resulted in larger absolute volume contractions in accord with earlier observations (Schagatay et al., 2015; Holmström et al., 2020), while there was no correlation between initial spleen volume and percentage of contraction, showing that individual spleen size is most important for the effect. Thus, individuals with large spleens likely have a greater potential to increase circulating Hb.

The Hb elevation at 4.1 g·L^−1^ was within values observed previously: 2.8, 3.5, and 5.4 g·L^−1^ during different eupneic hypoxic situations (Richardson et al., 2005, 2008; Schagatay et al., 2020a). While the average Hb response was 4.1 g·L^−1^ in the whole group, there was also a great individual variation in this response, with up to 12.3 g·L^−1^ Hb increase, and it would be valuable to evaluate this response in a larger sample to reveal differences in strong responders. The positive association between Hb increase and the reduction of SpO_2_ at the end of EXP could indicate a greater response in the most hypoxemic individuals; it agrees with the response-initiation by hypoxia and also seems functional as it elevates the arterial content of oxygen and maintains adequate oxygen delivery (Moraga et al., 2018). The Hb increase was, however, not associated with spleen volume or with the magnitude of spleen volume reduction, as found in some previous studies (Espersen et al., 2002; Richardson et al., 2005; Prommer et al., 2007; Engan et al., 2014). In Richardson et al. (2008), the spleen was estimated to contribute to 60% of the observed overall increase in hematocrit during hypoxic exposure, and thus, the increase in Hb was not only explained by spleen contraction, which may be the case also in this study. Perhaps, as in other animal species, humans can use another blood reservoir organ, e.g., liver and kidneys, when the arterial oxygen content is reduced (Carneiro and Donald, 1977; Xia et al., 2016). In any case, spleen contraction seems to be a major contributor to elevated Hb.

This study adds more information about the acute response of the spleen to hypoxia, which could apply to both exposure to extreme environments and some diseases and warrants further research to explain the underlying mechanisms and functional effects.

### Limitations

Measurement of Hb was done only after maximal exposure time, and it would have been informative to collect data also after 3 min when spleen contraction was already evident. The sample is small with great inter-individual variation, as often in this type of experimental studies, which makes correlation analyses unreliable. We did not study changes induced just by the stress of the protocol on the spleen volume, which would be useful for the determination of the details of the underlying mechanisms. Additionally, we did not measure plasma volume that could affect the values of Hb under hypoxia (Beidleman et al., 2017; Young et al., 2019; Schlittler et al., 2021), although we believe this short exposure could not induce significant changes responsible for the observations. In two participants, the hemoglobin concentration was measured on capillary samples that can yield higher results (Rappaport et al., 2020), although they acted as their own controls, and this should not affect the changes studied.

## Conclusion

We conclude that sudden eupneic hypoxia as short as 10 min caused significant spleen contraction leading to Hb increase, a response nearly twice as powerful as seen with slowly developing hypoxia. We speculate that this rapid spleen response, evident already after 3 min of exposure, could have a protective effect during sudden exposure to severe hypoxia in different situations. The response magnitude was highly individual, and we speculate that this could reflect different tolerance to sudden hypoxia.

## Data Availability Statement

The raw data supporting the conclusions of this article will be made available by the authors, if confidentiality is guaranteed.

## Ethics Statement

The studies involving human participants were reviewed and approved by Regional Human Ethics Board of Umeå University. The patients/participants provided their written informed consent to participate in this study.

## Author Contributions

FP contributed to the data analysis, interpretation, and manuscript writing. FS contributed to the conception of the study, data acquisition, preliminary analysis, and critical review of the manuscript. CV contributed to the data acquisition, preliminary analysis, and critical review of the manuscript. ES contributed to the conception of the study, data acquisition, data analysis, and manuscript writing. All authors contributed to the article and approved the submitted version.

## Funding

Funding was obtained from the Mid Sweden University and the Swedish Centre for Research in Sports (CIF).

## Conflict of Interest

The authors declare that the research was conducted in the absence of any commercial or financial relationships that could be construed as a potential conflict of interest.

## Publisher's Note

All claims expressed in this article are solely those of the authors and do not necessarily represent those of their affiliated organizations, or those of the publisher, the editors and the reviewers. Any product that may be evaluated in this article, or claim that may be made by its manufacturer, is not guaranteed or endorsed by the publisher.
